# Supplementary material for: Exploring the Relationship between Trichome and Terpene Chemistry in Chrysanthemum
Source: Plants (Basel). 2022 May 26;11(11):1410. doi: 10.3390/plants11111410 (PMC9182802; doi:10.3390/plants11111410)
Supplement: Supplementary file 1 [file plants-11-01410-s001.zip › plants-1722949-supplementary.pdf]

Table S1: Contents and emission rates of volatile terpenes compounds in flowerheads and leaves of 3 chrysanthemums cultivars

Compounds	Retention	CJ				GJB				HBJ			
	Index	Flower(C)	Leaf (C)	Flower (E)	Leaf (E)	Flower (C)	Leaf (C)	Flower (E)	Leaf (E)	Flower (C)	Leaf (C)	Flower (E)	Leaf (E)
**Monoterpene hydrocarbons**													
Tricyclene	934	ND	ND	7.93 ±0.44	ND	ND	ND	ND	ND	ND	ND	ND	ND
3-Carene	941	ND	ND	ND	ND	ND	83.19±0.71	49.13±4.18	ND	ND	ND	ND	ND
Bicyclo[3.1.1]hept-2-ene, 3,6,6-trimethyl-	942	ND	ND	54.51±4.45	ND	ND	ND	ND	ND	ND	ND	18.16±2.12	ND
β-Thujene	942	ND	ND	ND	ND	68.03±3.50	ND	ND	ND	91.35±2.26	ND	ND	ND
Cyclohexene, 4-methylene-1-(1-methylethyl)-	945	ND	ND	ND	ND	ND	ND	ND	ND	ND	ND	ND	ND
Camphene	954	ND	23.53±3.01	116.57±6.05	ND	ND	ND	12.02±1.82	ND	ND	ND	7.63±1.18	ND
β-Pinene	959	ND	ND	ND	ND	ND	ND	ND	ND	ND	ND	ND	ND
Sabinene	978	ND	ND	ND	ND	ND	ND	61.82±5.06	ND	ND	ND	29.16±0.60	ND
β-Phellandrene	1032	ND	ND	ND	ND	ND	158.03±17.28	ND	ND	ND	ND	ND	ND
**Oxygenated monoterpenes**													
γ-Terpinene	1061	ND	ND	ND	ND	ND	ND	ND	ND	ND	ND	ND	ND
1,3-Cyclohexadiene-1-carboxaldehyde, 2,6,6-trimethyl-	1025	ND	ND	ND	ND	ND	ND	ND	ND	43.74±1.62	ND	13.03±1.11	ND
4-Thujanol	1031	ND	ND	ND	ND	ND	ND	ND	ND	ND	ND	ND	ND
Eucalyptol	1033	173.49±5.28	78.98±1.32	113.61±3.69	ND	296.27±13.75	48.68±5.30	31.44±1.53	ND	126.85±6.57	21.83±2.11	30.45±1.12	ND ND

2,6,6-Trimethylbicyclo[3.2.0]hept-2-en-7-one	1064	ND	ND	ND	ND	ND	ND	ND	ND	100.43+2.59	ND	ND	ND
Thujone	1066	ND	ND	ND	ND	ND	ND	ND	ND	78.545±5.38	ND	ND	ND
Sabinene hydrate	1069	ND	ND	ND	ND	ND	ND	ND	ND	ND	ND	ND	ND
Isocyclocitral	1096	ND	ND	ND	ND	ND	ND	ND	ND	ND	ND	ND	ND
Camphor	1103	338.61±0.85	ND	87.18±2.23	ND ND	1130.27±67.91	ND	10.66 ±0.83	ND	ND	ND	8.28±1.7	ND
2-Bornanone	1147	ND	145.94±21.43	ND	ND	ND	184.24±18.28	ND	ND	124.50±7.84	ND	ND	ND
trans-Verbenol	1148	ND	ND	ND	ND	ND	ND	ND	ND	46.25±3.94	ND	ND	ND
cis-Chrysanthenol	1166	ND	ND	ND	ND	78.54±3.10	ND	ND	ND	100.57±5.91	ND	ND	ND
endo-Borneol	1168	349.02±13.20	154.15±19.69	50.26±0.53	ND	134.76±2.30	ND	6.41 ±0.49	ND	80.18±5.06	ND	10.66±2.12	ND
2-Cyclohexen-1-one,3-methyl-6-(1-methylethyl)-	1229	ND	ND	ND	ND	ND	ND	ND	ND	59.42±4.57	ND	25.55±2.24	ND
**Sesquiterpene hydrocarbons**													
γ-Elemene	1330	ND	ND	ND	ND	ND	30.79±1.57	ND	ND	ND	ND	ND	ND
Bicyclo[5.2.0]nonane, 2-methylene-4,8,8-trimethyl-4-vinyl-	1363	ND	ND	ND	ND	57.28±2.19	ND	ND	ND	ND	ND	ND	ND
β-Elemene	1391	ND	ND	ND	ND	ND	191.36±9.51	ND	ND	ND	ND	ND	ND
β-Caryophyllene	1422	ND	94.3±2.94	ND	ND	ND	311.19±30.51	ND	ND	ND	ND	ND	ND
β-Copaene	1431	57.33±1.50	32.66±2.67	ND	ND	ND	135.41±7.78	ND	ND	ND	32.87±1.43	ND	ND
Chrysantenyl 2-methuylbutanoate	1433	ND	ND	ND	ND	ND	ND	ND	ND	ND	ND	ND	ND

β-Selinene	1433	676.64±13.15	64.74±0.20	121.17±3.22	ND	ND	ND	ND	ND	ND	ND	ND	ND
α-Cedrene	1443	59.26±1.99	ND	ND	ND	ND	ND	ND	ND	ND	ND	ND	ND
cis-α-Bergamotene	1445	ND	ND	ND	ND	ND	ND	100.42±3.71	ND	ND	ND	ND	ND
γ-Muurolene	1458	ND	ND	ND	ND	ND	84.56±4.39	ND	ND	ND	36.51±1.41	ND	ND
Curcumene	1482	ND	140.51±2.63	ND	ND	ND	ND	ND	ND	ND	ND	ND	ND
cis-β-Copaene	1486	ND	111.6±1.07	ND	ND	ND	378.08±19.0	ND	ND	ND	140.69±14.15	ND	ND
cis-β-Farnesene	1489	ND	25.65±1.11	ND	ND	51.40±2.08	ND	ND	ND	ND	ND	ND	ND
α-Zingeberene	1499	ND	ND	ND	ND	ND	ND	ND	ND	58.57±1.14	452.68±28.52	ND	ND
Germacrene D	1505	ND	ND	ND	ND	ND	64.07±5.43	ND	ND	ND	17.13±0.98	ND	ND
α-Farnesene	1511	ND	ND	ND	ND	ND	71.93±5.14	ND	ND	ND	59.33±2.07	ND	ND
β-Sesquiphellandrene	1529	102.70±1.13	258.15±8.8	ND	ND	ND	ND	ND	ND	ND	39.63±0.96	ND	ND
(E)-γ-Bisabolene	1601	ND	53.69±3.33	ND	ND	ND	319.01±10.97	ND	ND	ND	48.39±1.40	ND	ND
**Oxygenated sesquiterpenes**													
Cubebol	1464	ND	ND	ND	ND	ND	ND	ND	ND	46.28±1.70	ND	ND	ND
Caryophyllene oxide	1588	ND	31.56±0.25	ND	ND	ND	ND	ND	ND	ND	ND	ND	ND
Neointermedeol	1659	ND	45.12±5.76	ND	ND	ND	ND	ND	ND	ND	ND	ND	ND
Selin-6-en-4.alpha.-ol	1663	124.86±1.43	ND	ND	ND	85.74±9.91	ND	ND	ND	65.34±2.14	ND	ND	ND
4(15),5,10(14)-Germacratrien-1-ol	1670	ND	105.02±7.2	ND	ND	ND	ND	ND	ND	ND	ND	ND	ND

Longifolenaldehyde	1729	ND	46.93±3.82	ND	ND	ND	ND	ND	ND	ND	ND	ND	ND
β-Oplopenone	1742	ND	ND	ND	ND	ND	ND	ND	ND	ND	ND	ND	ND
Kessane	1748	ND	ND	ND	ND	ND	ND	ND	ND	ND	ND	ND	ND
2-((2R,4aR)-4a,8-Dimethyl-1,2,3,4,4a,5,6,7-octahydronaphthalen-2-yl)prop-2-en-1-ol	1766	ND	ND	ND	ND	ND	ND	ND	ND	ND	ND	ND	ND
Acorenone B	1791	ND	ND	ND	ND	ND	ND	ND	ND	95.04±6.58	ND	ND	ND
15-Copaenol	1814	159.62±4.39	ND	ND	ND	ND	ND	ND	ND	ND	ND	ND	ND
cis-Z-α-Bisabolene epoxide	1850	ND	ND	ND	ND	ND	31.58±2.73	ND	ND	ND	ND	ND	ND

Table S2: Contents and emission rates of volatile terpenes compounds in flowerheads and leaves of 3 chrysanthemums accessions

Compounds	Retention	LSYJ				ZJSYJ				NJYJ			
	Index	Flower (C)	Leaf (C)	Flower (E)	Leaf (E)	Flower (C)	Leaf (C)	Flower (E)	Leaf (E)	Flower (C)	Leaf (C)	Flower (E)	Leaf (E)
**Monoterpene hydroarbons**													
Tricyclene	934	ND	ND	ND	ND	ND	ND	ND	ND	ND	ND	ND	ND
3-Carene	941	ND	298.60±15.92	ND	ND	ND	25.13±2.38	ND	ND	ND	ND	ND	ND
Bicyclo[3.1.1]hept-2-ene,3,6,6-trimethyl-	942	ND	ND	14.9±1.20	ND	ND	ND	83.41±9.01	ND	ND	ND	ND	ND
β-Thujene	942	57.21±0.86	ND	ND	ND	96.67±10.23	ND	ND	ND	88.68±5.89	ND	ND	ND
Cyclohexene, 4-methylene-1-(1-methylethyl)-	945	ND	ND	ND	ND	46.51±6.67	ND	ND	ND	ND	ND	ND	ND
Camphene	954	ND	ND	ND	ND	ND	ND	ND	ND	ND	245.73±2.94	111.78±4.40	ND
β-Pinene	959	60.69±1.87	ND	ND	ND	ND	ND	ND	ND	ND	ND	ND	ND
Sabinene	978	ND	153.27±2.95	7.46±1.86	ND	ND	ND	14.81±3.67	ND	ND	837.74±35.99	ND	ND
β-Phellandrene	1032	ND	ND	ND	ND	ND	ND	ND	ND	ND	ND	ND	ND
**Oxygenated monoterpenes**													
γ-Terpinene	1061	ND	82.24±5.61	ND	ND	ND	ND	ND	ND	ND	200.55±10.53	ND	ND
1,3-Cyclohexadiene-1-carboxaldehyde, 2,6,6-trimethyl-	1025	ND	ND	ND	ND	ND	ND	ND	ND	ND	ND	ND	ND
4-Thujanol	1031	ND	ND	ND	ND	ND	ND	ND	ND	ND	ND	ND	ND

Eucalyptol	1033		181.44±4.46	487.09±13.40	29.57±2.62	ND	255.69±18.58	ND	ND	ND	ND	305.88±44.41	57.66±1.09	ND
2,6,6-Trimethylbicyclo[3.2.0] hept-2-en-7-one	1064		ND	21.50±0.89	ND	ND	ND	ND	ND	ND	1318.51±30.55	ND	ND	ND
Thujone	1066		127.50±11.08	ND	ND	ND	ND	ND	ND	ND	ND	ND	ND	ND
Sabinene hydrate	1069		ND	ND	ND	ND	ND	ND	ND	ND	ND	84.08±5.57	ND	ND
Isocyclocitral	1096		ND	ND	ND	ND	43.33±1.73	ND	ND	ND	ND	ND	ND	ND
Camphor	1103		ND	ND	ND	ND	ND	ND	ND	ND	ND	ND	158.31±11.43	ND
2-Bornanone	1147		139.76±6.64	ND	7.31±0.30	ND	ND	ND	ND	ND	ND	882.34±15.29	ND	ND
trans-Verbenol	1148		113.18±5.92	378.36±4.41	ND	ND	230.79±9.72	68.72±4.74	ND	ND	ND	ND	ND	ND
cis-Chrysanthenol	1166		566.52±21.53	958.8±43.51	ND	ND	945.32±32.82	182.28±13.29	31.64±2.34	ND	ND	ND	ND	ND
endo-Borneol	1168		ND	ND	2.29±0.3	ND	ND	ND	ND	ND	ND	145.02±2.96	ND	ND
2-Cyclohexen-1-one ,3-methyl-6-(1-methylethyl)-	1229		ND	ND	ND	ND	ND	ND	ND	ND	ND	ND	ND	ND
**Sesquiterpene hydrocarbons**														
γ-Elemene		1330	ND	ND	ND	ND	ND	ND	ND	ND	ND	ND	ND	ND
Bicyclo[5.2.0]nonane, 2-methylene-4,8,8-trimethyl-4-vinyl-		1363	54.33±1.67	ND	ND	ND	101.18±3.88	ND	ND	ND	53.27±6.50	ND	ND	ND
β-Elemene		1391	99.71±3.12	327.52±4.81	ND	ND	177.92±5.62	356.06±29.09	ND	ND	52.79±1.10	ND	ND	ND
β-Caryophyllene		1422	ND	395.04±11.38	ND	ND	ND	351.97±9.64	3.05±0.1	ND	65.02±1.09	ND	ND	ND
β-Copaene		1431	ND	57.36±3.29	ND	ND	ND	82.13±7.85	ND	ND	ND	ND	ND	ND

Chrysantenyl 2-methuylbutanoate	1433	189.78±5.67	ND	ND	ND	ND	ND	ND	ND	ND	ND	ND	ND
β-Selinene	1433	ND	ND	ND	ND	ND	ND	ND	ND	27.89±0.89	ND	ND	ND
α-Cedrene	1443	ND	ND	ND	ND	ND	ND	ND	ND	ND	ND	ND	ND
cis-α-Bergamotene	1445	ND	ND	ND	ND	38.46±1.64	51.31±13.77	ND	ND	36.97±1.85	ND	ND	ND
γ-Muurolene	1458	ND	ND	ND	ND	ND	66.55±5.06	ND	ND	ND	ND	ND	ND
Curcumene	1482	ND	ND	ND	ND	ND	ND	ND	ND	ND	ND	ND	ND
cis-β-Copaene	1486	ND	157.89±7.2	ND	ND	ND	233.70±24.47	ND	ND	ND	ND	ND	ND
cis-β-Farnesene	1489	ND	ND	ND	ND	171.76±4.85	ND	ND	ND	ND	439.45±5.89	ND	ND
α-Zingeberene	1499	ND	ND	ND	ND	ND	ND	ND	ND	ND	ND	ND	ND
Germacrene D	1505	ND	ND	ND	ND	ND	33.32±2.64	ND	ND	ND	ND	ND	ND
α-Farnesene	1511	ND	ND	ND	ND	ND	101.75±9.23	ND	ND	ND	ND	ND	ND
β-Sesquiphellandrene	1529	ND	ND	ND	ND	ND	ND	ND	ND	ND	ND	ND	ND
(E)-γ-Bisabolene	1601	ND	ND	ND	ND	79.89±3.69	123.54±15.76	ND	ND	ND	ND	ND	ND
**Oxygenated sesquiterpenes**													
Cubebol	1464	ND	77.75±4.11	ND	ND	ND	ND	ND	ND	ND	ND	ND	ND
Caryophyllene oxide	1588	ND	96.86±7.86	ND	ND	ND	ND	ND	ND	ND	141.83±8.93	ND	ND
Neointermedeol	1659	ND	98.47±5.01	ND	ND	ND	ND	ND	ND	ND	97.61±1.75	ND	ND
Selin-6-en-4.alpha.-ol	1663	ND	ND	ND	ND	ND	ND	ND	ND	ND	79±3.83	ND	ND
4(15),5,10(14)-Germacratrien-1-ol	1670	ND	134.98±15.08	ND	ND	ND	ND	ND	ND	ND	238.55±13.41	ND	ND
Longifolenaldehyde	1729	ND	ND	ND	ND	ND	ND	ND	ND	ND	ND	ND	ND
β-Oplopenone	1742	ND	ND	ND	ND	ND	ND	ND	ND	ND	73.95±5.47	ND	ND
Kessane	1748	ND	107.92±11.92	ND	ND	ND	ND	ND	ND	ND	81.01±3.80	ND	ND
2-((2R,4aR)-4a,8-Dimethyl-1,2,3,4,4a,5,6,7-	1766	ND	ND	ND	ND	ND	104.07±1.56	ND	ND	ND	ND	ND	ND

octahydronaphthalen-2-yl)prop-2-en-1-ol													
Acorenone B	1791	ND	ND	ND	ND	ND	ND	ND	ND	ND	ND	ND	ND
15-Copaenol	1814	ND	ND	ND	ND	ND	ND	ND	ND	47.27±2.49	338.41±19.04	ND	ND
cis-Z-α-Bisabolene epoxide	1850	ND	ND	ND	ND	ND	ND	ND	ND	ND	ND	ND	ND

Note: ND represents not detected; Flower (C) and Leaf (C) represent accumulated concentration of terpenoid components in flower and leaf; Flower (E) and Leaf (E) represent emission rate of terpenoid compounds in flower and leaf; Mean ±SE, the Mean values were calculated with three biological repetition (relative values >1% of total amount).
